# Factors affecting fluoroscopy time during percutaneous nephrolithotomy: Impact of stone volume distribution in renal collecting system

**DOI:** 10.1590/S1677-5538.IBJU.2019.0111

**Published:** 2019-12-17

**Authors:** Sait Özbir, Hasan Anil Atalay, Halil Lütfi Canat, Mehmet Gökhan Çulha, Süleyman Sami Çakir, Osman Can, Alper Ötünçtemur

**Affiliations:** 1 Department of Urology, Okmeydani Training and Research Hospital, Şişli, Istanbul, Turkey

**Keywords:** Fluoroscopy, Nephrolithotomy, Percutaneous, Cakut [Supplementary Concept]

## Abstract

**Purpose:**

To identify the factors increased fluoroscopy time during percutaneous nephrolithotomy and investigate the relationship between the 3D segmentation volume ratio of stone to renal collecting system and fluoroscopy time.

**Materials and Methods:**

Data from 102 patients who underwent percutaneous nephrolithotomy were analyzed retrospectively. Volume segmentation of both the renal collecting system and stones were obtained from 3D segmentation software with the images on CT data. Analyzed stone volume (ASV), renal collecting system volume (RCSV) measured and the ASV-to-RCSV ratio was calculated. Several parameters were evaluated for their predictive ability with regard to fluoroscopy time.

**Results:**

The stone-free rate was 55.9% after the percutaneous nephrolithotomy. Complications occurred in 31(30.4%) patients. The mean fluoroscopy time was 199.4±151.1 seconds. The fluoroscopy time was significantly associated with the ASV-to-RCSV ratio (p<0.001, r=0.614). The single tract was used in 77 (75.5%) cases while multiple tracts were used in 25 (24.5%) cases. Fluoroscopy time was significantly associated with multiple access (p<0.001, r=0.689). On univariate linear regression analysis, longer fluoroscopy time was related with increased stone size, increased stone volume, increased number of access, increased calyx number with stone, increased ASV-to-RCSV, increased operative time and decreased stone essence. On multivariate linear regression analysis, the number of access and the ASV-to-RCSV were independent predictors of fluoroscopy time during percutaneous nephrolithotomy.

**Conclusions:**

The distribution of the stone burden volume in the pelvicalyceal system is a significant predictor for prolonged fluoroscopy time during percutaneous nephrolithotomy. Measures to decrease FT could be beneficial in patients with a high ASV-to-RCSV ratio for precise preoperative planning.

## INTRODUCTION

Percutaneous nephrolithotomy (PCNL) has become standard treatment for complex renal stones from when it was first portrayed by Fernström and Johansson in 1976 (1). Alltough it has some advantages such as high success, low morbidity and early convalescence, PCNL is still related to higher radiation exposure compared to other urological procedures (2–4).

Radiation exposure is a well-known risk factor for malignancies and this risk is linear with regard to duration of exposure (5). The surgeons evaluate the pelvicalyceal system and choose the optimal calyx for puncturing with the help of fluoroscopy. As a result, it is significant to identify the related factors that affect fluoroscopy time (FT) during PCNL. FT is an important factor used for the evaluation of radiation exposure during urological operations (4).

There are several studies investigating this relationship. Tepeler et al. found that FT was significantly prolonged in patients with large renal stones necessitating multiple accesses (6). Mancini et al. likewise found that stone burden, multiple access and higher Body Mass Index (BMI) were associated with increased radiation exposure (7). Noureldin et al. concluded that estimated blood loss (EBL), number of punctures and operative time were the independent predictors of prolonged FT (8). A recent study showed that FT was associated with stone burden, stone location, number of stones, number of punctures and number of tracts (9).

Three-dimensional (3D) volume segmentation method is an important part of diagnosis with computer-aided medical applications. In the present study, we examined the relationship between the 3D segmentation volume ratio of a stone to renal collecting system and FT. To the best of our knowledge, this is the first study investigating this relationship.

## MATERIALS AND METHODS

A retrospective study conducted after the approval of the Institutional Ethics Committee. From January 2013 to July 2018, 102 patients were chosen to the present investigation who met the criteria. Patients with anatomical anomalies and patients with incomplete data were excluded from the study. All procedures were implemented by a single experienced surgeon.

Our indications of PCNL medical procedure are guided by the proposals of EAU and AUA guidelines (10, 11). Laboratory examinations were noted. All patients experienced NCCT (Toshiba Alexion^™^ multislice CT). The perioperative and postoperative information including number of tracts, scopy time, operative time, stone-free status, hemoglobin drop, blood transfusion requirement, complications, length of stay (LOS) and second procedure necessity were additionally recorded. Flouroscopy time was characterized as the aggregate time for which fluoroscopy was utilized amid every methodology. Operative time was characterized as the length from the entrance of the targeted calyx to the fulfillment of the method. Hemoglobin drop was defined as the contrast amongst preoperative and 24-hour postoperative hemoglobin concentrations. Modified Clavien classification system was used to characterize complications after PCNL (12).

**Figure 1:**
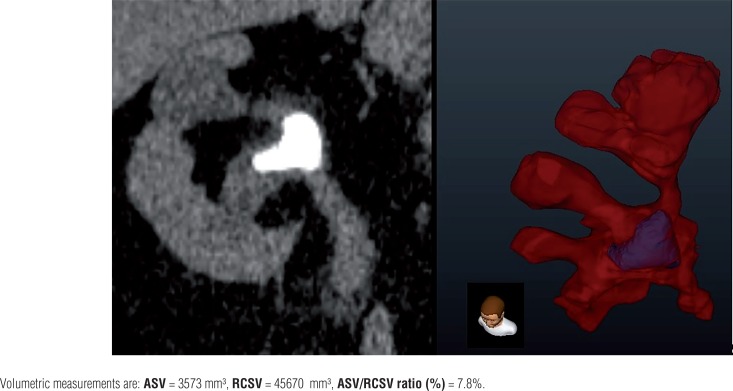
NCCT and 3D volume segmentation images of pelvicalyceal system and stones.

After the production of a 3D surface volume rendering of renal stones and the gathering framework, division of the renal collecting system volume (RCSV) and analyzed stone volume (ASV) of all patients delineating the pelvicalyceal anatomy and the volumetric stone weight dissemination in the gathering framework were analyzed (Dornheim Segmenter, Mainz, Germany). Images acquired from transverse NCCT images were utilized for volume division by using seed-based district developing technique. Images of the renal gathering framework from the NCCT images were chosen and utilizing a beginning stage, images were picked physically with the seed-based area developing the device. The division was actualized by thresholding strategy, a scope of qualities from the NCCT information of stones was chosen and information that fell outside the scope of limit values were rejected from the investigation (Figure-1). ASV and RCSV were estimated and the ASV-to--RCSV proportion was computed.

The procedure of PCNL used in our foundation was portrayed in our past investigations (13, 14). A 5 or 6F open-finished ureteral catheter was embedded in the lithotomy position. From there on, patients were put in the prone position. Percutaneous access was accomplished under fluoroscopic direction. Balloon dilators were utilized for tract dilatation. Fragmentation and stone expulsion were achieved utilizing pneumatic or ultrasound energy and recovery graspers through unbending 22F-26F nephroscopes. A 16F re-entry catheter or an 18F Foley catheter was embedded after the completion of the method. The patient was released the following day if the patient was comfortable, afebrile, and there was no drainage from the nephrostomy site after the nephrostomy tube was evacuated. Patients were assessed with respect to the sum and area of residual stone on the postoperative first day and following 3 months by utilizing imaging modalities (plain X-ray of the kidneys, ureters, and bladder (KUB) or on an NCCT if the stone was radiolucent). The Ziehm Solo 51026 mobile C-arm with automatic exposure settings (kV/mA, brightness) were utilized to implement low-dose continual fluoroscopy with a rate of 30 pulses /sec. Settings of fluoroscopy were constant for all patients. Fluoroscopy utilization was self-controlled by the surgeon with a foot pedal while the C-arm manually was controlled by an operator under surgeon's directions.

The study was conducted as a retrospective cohort. Spearman rank correlation coefficient or Pearson's correlation coefficient were used for assessing the relationship between continuous variables. Univariate and multivariate linear regression analyses were used to determine the predictors of FT. The data were analyzed with the Statistical Package for Social Sciences (SPSS) version 22.0^™^ (IBM Corporation, California). A two-tailed p-value <0.05 was considered statistically significant.

## RESULTS

**Figure 2:**
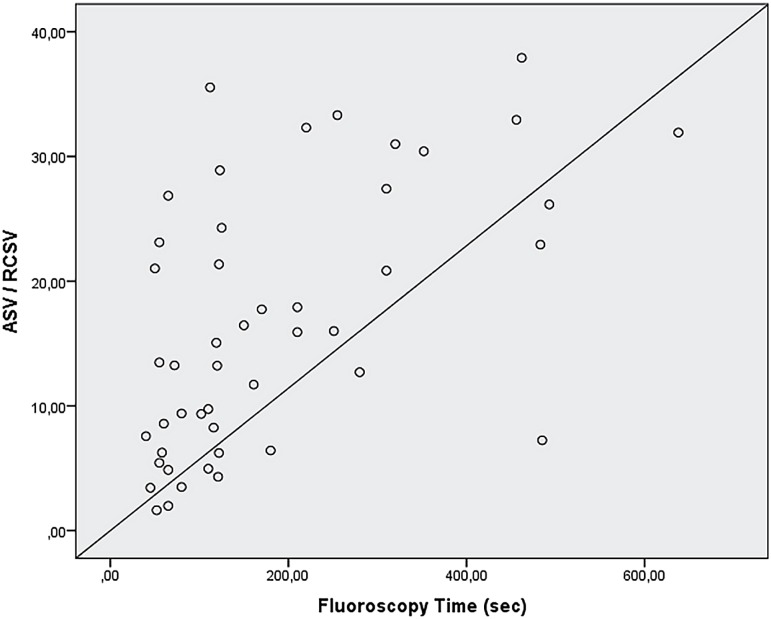
Correlation between ASV-to-RCSV ratio and Fluoroscopy time.

**Figure 3:**
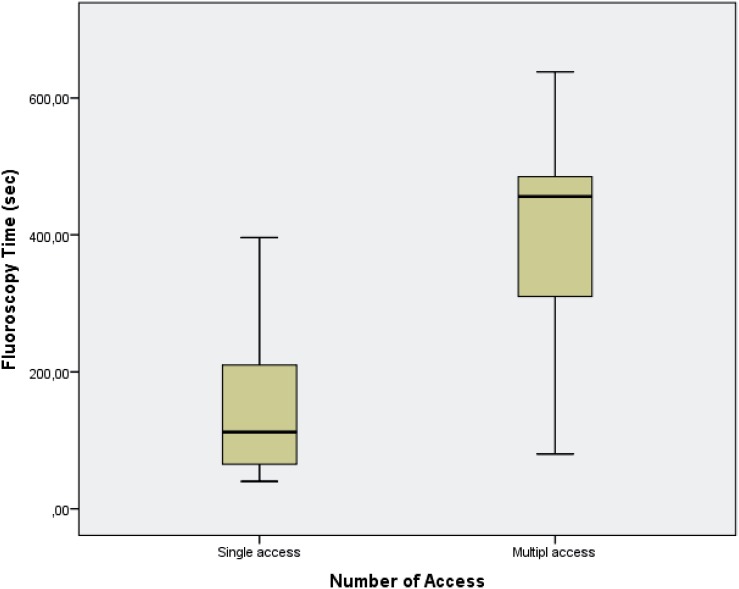
Effect of multi access on Fluoroscopy time.

For the final analysis, 102 patients were selected to the study. Clinical characteristics are shown in Table-1. The stone-free rate was 55.9% after the PCNL monotherapy. Complications occurred in 31 (30.4%) of the cases. Grade 1 complication occurred in 17 (16.7%) patients, grade 2 in 6 (5.9%) patients, grade 3a in 6 (5.9%) patients and grade 3b in 2 (2.0%). The mean length of stay (LOS) was 2.4±0.7 days. Mean FT was 199.4±151.1 seconds. As it is shown in Figure-2, FT was significantly associated with the ASV / RCSV ratio (p <0.001, r=0.614). FT was also correlated with stone size (p <0.001, r=0.543), number of stones (p=0.004, r=0.279). FT was significantly and positively correlated with operation time (p <0.001, r=0.643). FT was higher in patients with longer hospitalization time (p=0.004, r=0.444). The single tract was used in 77 (75.5%) cases while multiple tracts were used in 25 (24.5%) cases (Figure-3). FT was significantly associated with multiple access (p <0.001, r=0.689).

**Table 1:** Demographic, preoperative and perioperative features of study cohort.

		Patients
		Mean±S.D (n%)
Age		**46.2±13.5**
**Sex**		
	Male	57 (55.9%)
	Female	45 (44.1%)
**Side**		
	Right	48 (47.1%)
	Left	54 (52.9%)
BMI		26.2±3.4
Creatinine(mg/dL)		1.0±0.2
Tract length (mm)		81.9±22.5
Calyx no.with stone		3.4±1.1
Stone size (mm)		38.0±11.7
Stone density (HU)		1093.9±335.2
ASV (mm^3^)		627.9±600.6
RCSV(mm^3^)		4919.6±5264.4
ASV/RCSV(%)		17.7±10.7
**Access no.**		
	Single tract	77 (75.5%)
	Multiple tract	25 (24.5%)
Operation time (min.)		90.2±29.9
Access time (min.)		17.3±8.6
Fluoroscopy time (sec.)		199.4±151.1
Length of stay (LOS)		2.4±0.7
Hemoglobine drop (mg/dL)		1.7±1.1
**Transfusion**		
	Yes	14 (13.7%)
	No	88 (86.3%)
**Stone-free**		
	Yes	57 (55.9%)
	No	45 (44.1%)
**Modified Clavien Grade**		
	0	71 (69.6%)
	1	17 (16.7%)
	2	6 (5.9%)
	3a	6 (5.9%)
	3b	2 (2.0%)
	4a	0 (0.0%)
	4b	0 (0.0%)
	5	0 (0.0%)

**BMI** = body mass index; **HU** = Hounsfield unit; **ASV** = analyzed stone volume; **RCSV** = renal collecting system volume

On univariate linear regression analysis, longer FT was related with increased stone size, increased stone volume, increased number of accesses, increased calyx number with stone, increased ASV-to-RCSV, increased operative time and decreased stone essence. On multivariate linear regression analysis, the number of accesses and ASV-to-RCSV were independent predictors of FT during PCNL (Table-2).

**Table 2:** Predictors of fluoroscopy time on linear logistic regression analysis.

	Univariate model		Multivariate model	
	B Coefficient	p	B Coefficient	p
Age	0.3	0.49		
Sex(Male gender)	18	0.27		
Side (Right side)	25	0.40		
BMI(kg/m^2^)	3.5	0.42		
Tract length (mm)	0.6	0.65		
Calyx no.with stone	30	**<0.001**	1.7	0.95
Stone size (mm)	6.9	**<0.001**	1.6	0.25
Stone density (Radiolucent)	0.98	**<0.001**	0.1	0.43
ASV (mm^3^)	0.1	**<0.001**	0.1	0.42
RCSV(mm^3^)	−0.1	0.46		
ASV/RCSV(%)	8.5	**<0.001**	5.2	**<0.001**
Access no.(Multiple access)	167	**<0.001**	165	**<0.001**
Operation time (min)	3.2	**<0.001**	0.2	0.61
	2.7	0.87		
Post-operative Nephrostomy Tube	12	0.29		

## DISCUSSION

PCNL is the most appropriate treatment choice for renal stones larger than 2cm (10). Fluoroscopic guidance is also an important part of PCNL. However, not only the surgeon but also the patient and operating room staff have an increased risk of exposure with ionizing radiation. The effective radiation dose during PCNL is around 7-9mSv (7). International Commission on Radiological Protection (IRCP) recommends for occupational exposure a 20mSv safety limit yearly, and the effective dose should not exceed 50mSv in a single year (15). Surgeon experience and attitude are some of the crucial changeable factors for the duration of this exposure during the procedure. Radiation exposure during the procedure depends on several factors such as FT, distance and the properties of the fluoroscopy machine (pulse rate, KVP, mAs) (16, 17). FT is controllable, monitorable, easy and linear related to radiation exposure (9, 17). Therefore, several studies used FT as a proxy tool for estimating radiation doses and investigated the predictors that affect FT during PCNL (6–9).

The purpose of the current study was to search the factors that could predict increased FT. Our results demonstrated that ASV-to-RCSV and multiple access were the independent predictors for increased FT during PCNL. Our findings were congruent with previous reports. Multiple access has been well identified as a parameter increasing FT. Previous studies reported that multiple access and stone burden were associated with increased FT (6, 7). Moreover, a recent study showed that as soon as more than 2 attempts were required for puncturing the chosen calyx, FT was significantly higher (9). Therefore, single access rather than multiple access should always be the desired goal to decrease radiation exposure. Likewise, Noureldin et al. found that number of punctures, EBL and operative time were the only independent predictors of prolonged FT during PCNL whereas there was no correlation of FT with S.T.O.N.E. Nephrolithometry Score (8). Sfoungaristos et al. found that FT was associated with stone burden, stone location, number of stones, number of punctures and number of tracts. Furthermore, all scoring systems such as Guy's stone score, S.T.O.N.E. nephrolithometry, and CROES nomogram could predict the complex cases and the need for increased FT (9).

The 3D printed technology is a novel technology and has various applications in renal procedures. Furthermore, this technology is quite precise to demonstrate the anatomy of the renal collecting system. Previous studies illustrated the importance of 3D technology, especially, in the presurgical planning for kidney surgery. Atalay et al. reported that generating kidney models of PCSs by using 3D printing technology is feasible, and understandings of the disease and the surgical procedure from patients were well appreciated with this novel technology (18). Nevertheless, some other studies showed the importance of the renal anatomy applied to training and execution of the flexible ureteroscopy (19, 20). Recently, Marroig et al. standardized the building of a three-dimensional silicone mold (cavity) of the collecting system, on the basis of polyester resin endocasts, which can be used in surgical training programs (21).

As seen above, there is a controversy regarding the impact of nomograms on FT. These conflicting results may be enlightened with further studies. In the present study, there was no knowledge with regard to nomograms. Nevertheless, stone size, number, and location are the important variables that shared in common these scoring systems. Our previous studies illustrated that these variables may be better measured and analyzed with the 3D volume segmentation method (13, 14). Furthermore, hydronephrosis can be measured as a quantitative value. Therefore, the 3D volume segmentation method could increase the predictive power of nomograms. Additionally, stone branching and secondary calyceal stones might require multiple tracts (22). Likewise, Mishra et al. found that the need for tracts and stages were clearly established using the stone volume calculations by staghorn morphometry (23). Our study showed that the ASV-to-RCSV ratio and the number of tracts were the independent predictors for increased FT during PCNL. ASV-to-RCSV ratio is involved in the stone burden and the stone complexity. Additionally, the need for multiple tracts rises when the ASV-to-RCV ratio grows. Based on this, ASV-to-RCSV ratio might be a tool for the urologist before PCNL for assisting treatment planning and patients counseling. Likewise, our previous studies revealed that the ASV-to-RCSV ratio could predict outcomes of PCNL (13, 14).

In our study, the stone volume and the RCSV were calculated separately using the 3D volume segmentation method and the ratio between these variables was stated as a numerical value. As a result, the 3D segmentation method could be an alternative tool for more accurate numerical values for analyzing the renal collecting system and stone relation. ALARA (As Low As Reasonably Achievable) principles represents a practice keeping radiation doses to patients and personnel As Low As Reasonably Achievable (15, 24). The three major components of ALARA are time, distance and shielding (15, 24). Into the clinical practice, we can report that the ASV-to-RCV ratio may predict increased FT and be used to minimize the radiation exposition with the help of ALARA principles during PCNL.

A retrospective study design and relatively small sample size are some limitations of the current study. A prospective and large sample study could improve our results.

## CONCLUSIONS

FT rises with an increasing ASV-to-RCSV ratio for PCNL. In addition, the need for multiple tracts rises when the ASV-to-RCV ratio grows. The dispersion of the stone volume in the renal collecting system is an important predictor for prolonged FT during PCNL. Measures to decrease FT could be beneficial in patients with a high ASV--to-RCSV ratio for precise preoperative planning.

## ABBREVIATIONS

ASV: Analyzed stone burden volume
ALARA: As Low As Reasonably Achievable
BMI: Body mass index
CT: Computed tomography
DICOM: Digital imaging and communications in medicine
FT: Fluoroscopy time
PCNL: Percutaneous nephrolithotomy
RCSV: Renal collecting system volume
3D: Three dimensional
